# Computer-Aided Breast Surgery Framework Using a Markerless Augmented Reality Method

**DOI:** 10.3390/diagnostics12123123

**Published:** 2022-12-11

**Authors:** Seungwoo Khang, Taeyong Park, Junwoo Lee, Kyung Won Kim, Hyunjoo Song, Jeongjin Lee

**Affiliations:** 1 School of Computer Science and Engineering, Soongsil University, 369 Sangdo-ro, Dongjak-gu, Seoul 06978, Republic of Korea; 2 Department of Biomedical Informatics, Hallym University Medical Center, 22 Gwanpyeong-ro 170beon-gil, Dongan-gu, Anyang-si 14068, Republic of Korea; 3 Ewha Womans University Mokdong Hospital, 1071 Anyangcheon-ro, Yangcheon-gu, Seoul 07985, Republic of Korea; 4 Department of Radiology and Research Institute of Radiology, Asan Medical Center, University of Ulsan College of Medicine, 88 Olympic-ro, 43-gil, Songpa-gu, Seoul 05505, Republic of Korea

**Keywords:** medical imaging, augmented reality, markerless AR, 3D breast CT

## Abstract

This study proposes a markerless Augmented Reality (AR) surgical framework for breast lesion removal using a depth sensor and 3D breast Computed Tomography (CT) images. A patient mesh in the real coordinate system is acquired through a patient 3D scan using a depth sensor for registration. The patient mesh on the virtual coordinate system is obtained by contrast-based skin segmentation in 3D mesh generated from breast CT scans. Then, the nipple area is detected based on the gradient in the segmented skin area. The region of interest (ROI) is set based on the detection result to select the vertices in the virtual coordinate system. The mesh on the real and virtual coordinate systems is first aligned by matching the center of mass, and the Iterative Closest Point (ICP) method is applied to perform more precise registration. Experimental results of 20 patients’ data showed 98.35 ± 0.71% skin segmentation accuracy in terms of Dice Similarity Coefficient (DSC) value, 2.79 ± 1.54 mm nipple detection error, and 4.69 ± 1.95 mm registration error. Experiments using phantom and patient data also confirmed high accuracy in AR visualization. The proposed method in this study showed that the 3D AR visualization of medical data on the patient’s body is possible by using a single depth sensor without having to use markers.

## 1. Introduction

Breast cancer (BC) is the most common cancer and also the leading cause of cancer-related deaths in women, with the highest incidence (24.2%) and mortality (15%) of all female cancer patients worldwide. In particular, the BC incidence among Korean women is the highest among Asian countries [1]. Accordingly, there is an increasing demand for surgical procedures for breast lesion removal. The increasing demand for BC surgery has led to active research on various technologies for guiding surgical procedures, which can provide adequate intraoperative provision of the necessary information. In particular, Augmented Reality (AR) technology allows for the integrated visualization of 3D information by superimposing 3D information onto a real environment, thus providing a sense of reality and immersion as well as additional information [2,3]. Based on these strengths, research has been underway for various AR-based surgical navigation cases with improved precision. Previous studies on AR-based surgery include spine surgery [4], liver surgery [5], laparoscopic surgery [6], and bone tumor surgery [7]. These surgeries mainly used marker-based guidance, which has several limitations and challenges [8]. To overcome these limitations, there is an increasing need for non-marker-based AR research based on automated segmentation and registration of the region of interest (ROI) from medical images such as CT and MRI scans.

The representative previous studies related to the segmentation of the body region of a patient in a CT image are as follows: Zhou et al. [9] and Kang et al. [10] performed body region segmentation by using the automatic global thresholding technique based on Otsu’s method and applied the Connected Component Labeling (CCL) technique to remove unnecessary elements outside the body, such as the pad of CT equipment. Since these methods do not consider phase information and only consider the size of the segmented area, an error may occur depending on the range of the CT scan. Perez-Carrasco et al. [11] performed body region segmentation by randomly selecting some voxels with Hounsfield values of over 1700 in the CT images (those corresponding to bone) and, with these selected voxels as the seed, a neighborhood-connected region growing algorithm is applied. The selected voxels were used as the seed area, enabling the simple segmentation of the body region without considering other unnecessary factors. However, this approach has a disadvantage in that the range of HU values used for applying the region-growing technique must be manually input by the user.

Prior studies on feature-based registration using point sets acquired from the target for registration are as follows. Besl and McKay [12] proposed the Iterative Closest Point (ICP) technique. In this technique, for a predefined overlapping region between the two point sets given by input, the correspondence between the closest pair of points is determined, and iterative operations are performed to minimize the distance between them to derive the transformation matrix. Although the ICP technique has the advantage of low computational complexity while ensuring accurate registration, there is a problem that the computation load can increase rapidly as the number of points increases. In order to improve the pairwise registration error between points, Pulli [13] searched for points that are within a distance of a given threshold and applied additional constraints derived from experiments to the conventional ICP technique, such as allowing points to match only if their associated normal vectors differ by less than 45 degrees. However, since the threshold is set manually, their drawback is that the threshold value needs to be derived by conducting experiments multiple times. Zhang et al. [14] efficiently performed the process of searching for a pair of closest points by using a (k-dimensional) KD tree that is a generalization of a Binary Search Tree (BST) to a multidimensional space. Still, the disadvantage is that it is greatly affected by the initial position of the point set and noise.

In this study, we propose a markerless AR surgical framework using the mesh on the real coordinate system acquired through a 3D scan of the patient and the mesh on the virtual coordinate system acquired from 3D breast CT. The workflow of the proposed method is as follows. First, using Structure Sensor Mark II [15], the patient mesh in the real coordinate system is acquired through a 3D scan of the patient. Then, contrast-based skin segmentation (area under the skin) is performed on the 3D breast CT image. By using the Marching Cube technique [16] for the segmented skin area, skin mesh is generated, and in this way, the patient mesh in the virtual coordinate system is acquired. Considering the real-world setting in the operating room, the ROI is set based on the nipple position obtained through gradient-based search, and the vertices to be used for actual registration are selected. The method of 2.5-dimensional lesion segmentation through the propagation of the 2D segmented region allows fast and accurate lesion segmentation. The registration between the mesh in the real coordinate system and that in the virtual coordinate system is performed using the ICP technique [12] to the position where the distance difference between vertices is minimized. By applying the transformation factor obtained through registration to CT or lesion mesh, 3D integrated visualization of medical information by superimposition onto the patient’s body in real space is achieved. The proposed technique enables fast and accurate AR information while minimizing the patient’s burden by avoiding employing the markers.

The structure of this study is organized as follows. Section 2 describes the markerless AR surgical framework proposed in this study. Section 3 presents the experimental setting data and results, and Section 4 presents the conclusion of this study.

## 2. Methodology

**Figure 1:**
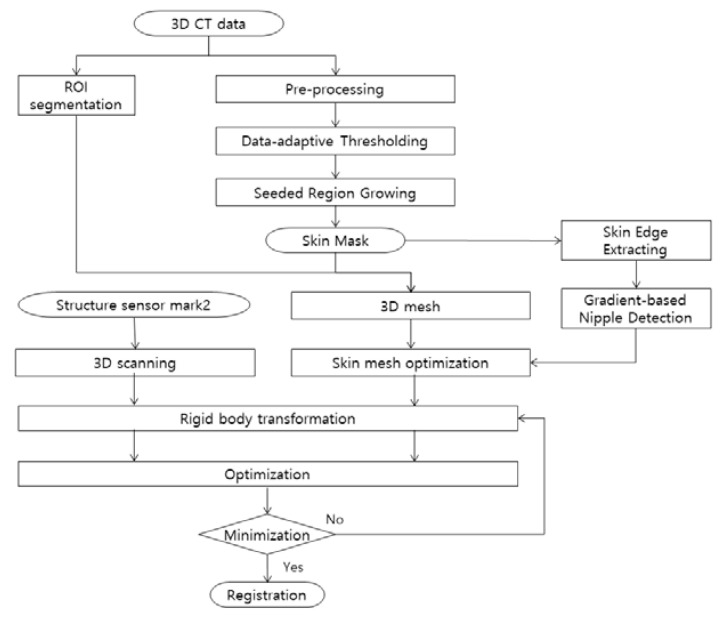
Markerless AR surgical framework.

In this study, we propose a markerless AR surgical framework, as shown in Figure 1 to derive the transformation relationship between a 3D breast CT scan taken before surgery and a patient in the real coordinate system without using a marker.

For registration between the patient in the real coordinate system and the CT image in the virtual coordinate system, (1) the Structure Sensor Mark 2 [15] is used to perform a 3D scan of the patient so that the patient mesh $M_{p}$ is acquired on the real coordinate system; (2) through body region (skin) segmentation of the CT image and generation of 3D mesh, mesh $M_{c}$ on the virtual coordinate system is acquired and used for registration. Since both meshes correspond to the same patient and the patient maintains similar postures, it is assumed that the shape difference between them is insignificant. In addition, by performing mesh optimization considering the memory and computational load and vertex selection through setting ROI reflecting the surgical environment, unnecessary computational load is reduced, and more robust registration is achieved. The 2.5-dimensional lesion segmentation technique proposed in this study enables fast and accurate lesion segmentation, which can be used to provide AR-based information through the application of registration information. The registration process involves deriving the point where the residual error between $M_{p}$ and $M_{c}$ present in the two respective coordinate systems is minimized. In this way, convergence to the optimal position is obtained.

### 2.1. Contrast-Based Skin Segmentation

CT scans may generate noise during the process of imaging or reconstruction. This results in errors in skin segmentation results. Therefore, in this study, an Anisotropic Diffusion Filter (ADF) technique [17], such as Equation (1), is capable of effectively reducing noise and maintaining edge information is applied prior to the process of skin segmentation.

(1) $$I_{i,\;j}^{t+1}=I_{i,\;j}^{t}+\lambda \left[C_{N}\;\cdot \;\nabla_{N}I+C_{S}\;\cdot \;\nabla_{S}I+C_{E}\;\cdot \;\nabla_{E}I+C_{W}\;\cdot \;\nabla_{W}I\right]$$

where $I_{i,\;j}^{t}$ means the pixel value of $\left(i,\;j\right)$ coordinates at time t, and N, S, W, E are the upper and lower, left and right directions. $\nabla_{N\sim E}I$, $C_{N\sim E}$ are the first derivation and transfer coefficient for pixel values in each direction. λ is a constant that determines the amount of change in a pixel value and must satisfy 0≤λ≤0.25.

Then, as shown in Equation (2), Otsu’s Method [18], in which the threshold value is automatically set in a data-adaptive manner based on the analysis of the diffusion of the pixel gray level value, is applied.

(2) $$\sigma_{\omega }^{2}=\omega_{0}\sigma_{0}^{2}+\omega_{1}\sigma_{1}^{2}\;\sigma_{Τ}^{2}=\omega_{0}{\left(\mu_{0}-\mu_{Τ}\right)}^{2}+\omega_{1}{\left(\mu_{1}-\mu_{Τ}\right)}^{2}=\omega_{0}\omega_{1}{\left(\mu_{1}-\mu_{0}\right)}^{2}$$

where ω, $\sigma^{2}$ and μ represent the weight, variance, and average pixel values, respectively, and Τ represents the threshold for classifying the patient region and background. Otsu’s Method [18] divides the patient region and the background into 1 and 0, and at this time, the patient region is used as the result of initial skin segmentation.

In the case of the thresholding-based segmentation technique, there is a problem in that the phase information of the image is not considered. In order to take into account the phase information and to remove the mis-segmented regions, the Seeded Region Growing (SRG) method [19], as shown in Equation (3) and the morphological operations are performed one after another.

(3) $$Τ=\left\{x\notin \overset{n}{\underset{i=1}{\cup }}A_{i}|N\left(x\right)\cap \overset{n}{\underset{i=1}{\cup }}A_{i}\neq \emptyset \right\}$$

$A_{i}$ denotes the initial n seed points, and $N\left(x\right)$ denotes a set of neighboring pixels adjacent to x in eight directions. For a set $N\left(x\right)$ satisfying x∈Τ, if $A_{i}$ or a region of $A_{i}$ is included—whether to include it is determined through a similarity comparison of pixel values. This process is repeated until similar pixels no longer exist nearby. In breast CT, the patient region is located at the center of the image. Therefore, by applying the SRG technique [19] by using arbitrary points in the patient region present in proximity to the center of the image as seed points, unnecessary elements such as pads of the CT equipment can be removed from the initial segmentation result. Then, by subsequently performing the SRG technique [19] with an arbitrary point in the background located at the top or bottom of the image as the seed point, the hole-filling effect can be obtained for the area inside the patient region. In addition, morphological operations that sequentially perform erosion and dilation are applied to preserve the shape and size of the skin while removing the mis-segmented area caused by noise, thereby improving the accuracy of skin segmentation results. The skin segmentation process is performed for all slices in each slice unit of the 3D breast CT given as the input, and the segmented skin regions are used for skin mesh generation.

### 2.2. Gradient-Based Nipple Detection

To calculate the gradient on the body surface, the gradient between two adjacent points is calculated as in Equation (4) for the edge of the skin segmentation region.

(4) $$\theta =atan\left(\frac{y_{2}-y_{1}}{x_{2}-x_{1}}\right)$$

At this time, the gradients of neighboring points are used together to minimize errors that may occur during the image-capturing process or segmentation process. By searching for gradient values calculated at consecutive points on the edge, the section where the amount of change shifts from increment to decrement is defined as the nipple candidate region. When there are two or more nipple candidate regions on one or both sides, the nipple region is finally selected using symmetry information. In each candidate region, a virtual candidate region is created so that nipple candidate regions can exist in pairs with reference to the center of the skin segmentation region. Then, the sum of gradient differences is calculated based on the region with the largest height, and the pair with the largest value of the sum is selected as the final nipple region.

### 2.3. 3D Skin Mesh Generation and Optimization

The skin region segmented from the entire slice is used to generate skin mesh through the Marching Cube technique [16]. Aliasing may occur in the generated mesh due to the characteristics of the Marching Cube technique that creates a polygonal mesh of an isosurface and the slice thickness of CT. This impacts the registration accuracy with 3D scan data and may result in heterogeneity in AR visualization. A decrease in the grid size of the Marching Cube can reduce the aliasing caused by the mesh generation process; however, this solution is insufficient for reducing aliasing caused by the slice thickness of CT in an image. Slice interpolation can be applied to solve issues that arise from slice thickness. 

However, the interpolation process may increase the number of slices, possibly leading to a significant increase in the amount of unnecessary calculation and memory use. To address this problem, smoothing using a Gaussian filter [20] is applied in the process before mesh generation. In addition, in the process of registration and visualization, decimation is performed to reduce the number of faces and vertices constituting the mesh to consider the computation load and memory utilization. In this case, the Fast-Quadric Mesh Simplification technique [21], which minimizes the shape deformation of the model and allows reducing the number of faces to meet the target value quickly, is applied. A tablet PC, providing comparatively insufficient resources, is used to conduct follow-up registration and AR visualization processes. For this reason, its performance related to calculation and memory should be considered to ensure a seamless AR environment. 

**Figure 2:**
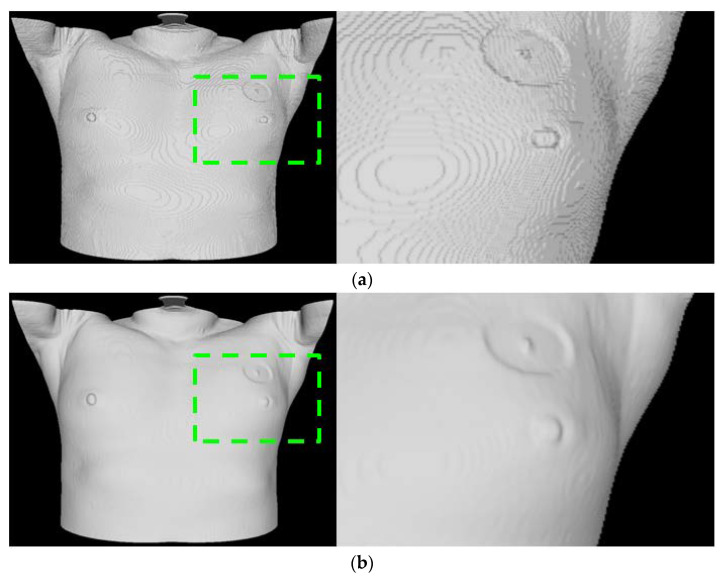
Results of skin mesh generation. (**a**) Skin mesh generated using Marching Cube algorithm. (**b**) Results obtained by application of Gaussian filtering and decimation.

Moreover, as the site of surgery (the left breast or the right breast) is exposed during surgery, using the entire skin mesh area obtained by CT may cause problems that can affect the registration process, such as getting stuck in local minima. To overcome this limitation, a vertex, practically applicable in the registration process, should be selected by determining the ROI based on the location of a nipple detected in the previous process. Based on reduced calculations, the vertex selection process facilitates a robust registration process that reflects the surgery environment. (Figure 2 presents the comparison result for the skin mesh generation).

### 2.4. ICP-Based Mesh Registration

ICP-based registration between $M_{p}$ obtained through a 3D scan of the patient and $M_{c}$ obtained from CT is performed. In addition, since both $M_{p}$ and $M_{c}$ are data of the same patient, the effect of scale is not considered. For the registration of the two meshes, the position is first aligned by matching the center of mass. Then, precise registration is performed using the Iterative Closest Point (ICP) technique [12]. The ICP method [12] is one of the most commonly used registration techniques because of its intuitive characteristic and low computational complexity. The ICP method uses the Euclidean distance to determine the correspondence between points at the shortest distance. Through rotations and translations, iterative operations are performed until the distance between the corresponding points in the matched pairs converges to a minimum. The transformation factor obtained as a result of the registration is applied to the CT image or ROI, such as the segmented lesion, to obtain visualization of additional information through AR on the patient’s body.

### 2.5. Segmentation of Lesion Area

**Figure 3:**
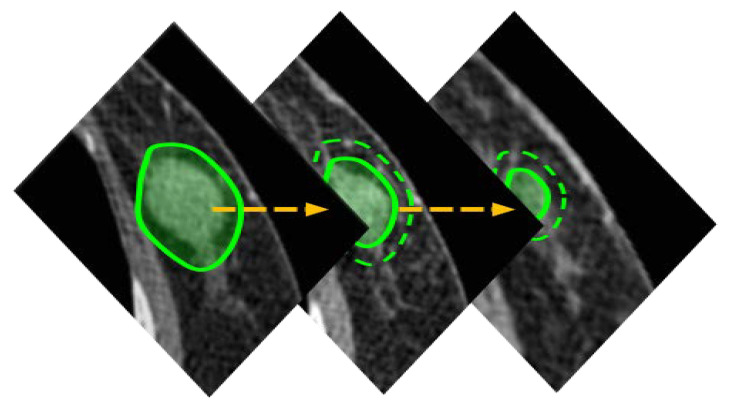
Process of seed region propagation.

To provide information on lesions as AR, segmentation of the applicable area needs to be preceded. However, segmenting a lesion in a medical image is highly challenging due to the image quality and the influence of neighboring tissues or organs. Identifying lesions on brain CT scans is notably difficult. Therefore, seed points are given as input to conduct the SRG method [19] as described above to generate a 2D segmentation result. By applying post-processing iterative morphological operations to the 2D segmentation result, the mis-segmented regions are removed due to reasons such as noise, and hole-filling is performed for the inside of the segmented region. The result of 2D segmentation is propagated to adjacent slices using the distance map [22] and used as a seed region for applying the SRG technique [19] in each slice. Then, the subsequent process is iterated. Figure 3 shows the simplified representation of the process of propagation. The green region indicates the propagation area, and the yellow arrow indicates the propagation steps. Propagation is performed until there are no more segmentation regions or until the first or last slice is reached in the process.

## 3. Experiments and Results

The experiments for the skin segmentation and nipple detection algorithms were performed using a PC with an Intel Core i5-2500 CPU (3.4GHz), RAM 32GB, Windows 7 64-bit, and Visual Studio 2012. The ICT-based registration algorithm was run on an Apple iPad Pro 11 A2228 [23], and the Occipital Structure Sensor Mark 2 [15] was used as a depth sensor via an Apple iPad Pro 11 A2228 and a bracket. The Occipital Structure Sensor Mark 2 is a commercial infrared depth sensor based on simultaneous localization and mapping (SLAM). This sensor provides necessary functions for AR visualization, such as calibration, 3D scanning, and object tracking.

### 3.1. Evaluation of Skin Segmentation Accuracy

The accuracy of skin segmentation was evaluated using breast CT data of 20 patients who had skin regions segmented manually by an expert. The accuracy of the skin segmentation results using the proposed method was evaluated by measuring the Dice Similarity Coefficient (*DSC*) with the reference region manually segmented by a specialist.

(5) $$DSC=\left(\frac{2\left|X\cap Y\right|}{\left|X\right|+\left|Y\right|}\right)$$

In this case, *X* and *Y* indicate a set of pixels belonging to the region obtained by the proposed technique and the region obtained by the specialist’s manual segmentation, respectively. The accuracy of the entire 3D data region is measured. The accuracy of the proposed skin segmentation method is presented in (Table 1), and the average accuracy is about 98.35 ± 0.71%, indicating very high accuracy of the result obtained by the proposed method.

### 3.2. Evaluation of Nipple Detection Accuracy

The accuracy of nipple area detection was evaluated using breast CT data of 20 patients whose nipple positions were entered manually by a specialist. The nipple positions entered by the specialist are composed of the x, y, and z coordinates of the position corresponding to the center on the boundary in the middle of the slice where the nipple is present for each of the left and right nipples. The error was calculated by measuring the three-dimensional Euclidean distance from the coordinates derived by the proposed method.

(6) $$\sqrt{{\left(x_{2}-x_{1}\right)}^{2}+{\left(y_{2}-y_{1}\right)}^{2}+{\left(z_{2}-z_{1}\right)}^{2}}$$

For the accuracy of the nipple detection method, the error in each data was measured based on the average value of the errors in the left and right nipples. The result of the accuracy analysis is shown in (Table 1), and the average of the measured errors is about 2.79 ± 1.54 mm.

### 3.3. Evaluation of Registration Accuracy

To evaluate registration accuracy, registration error was measured using phantom data and data of actual patients, respectively. For calculation of error, Fiducial Registration Error (FRE) between $M_{p}$ obtained by 3D scan and the skin mesh $M_{c}$ was obtained, and the number of faces of the skin mesh to be generated for registration was set to 192,000 for the experiment.

**Figure 4:**
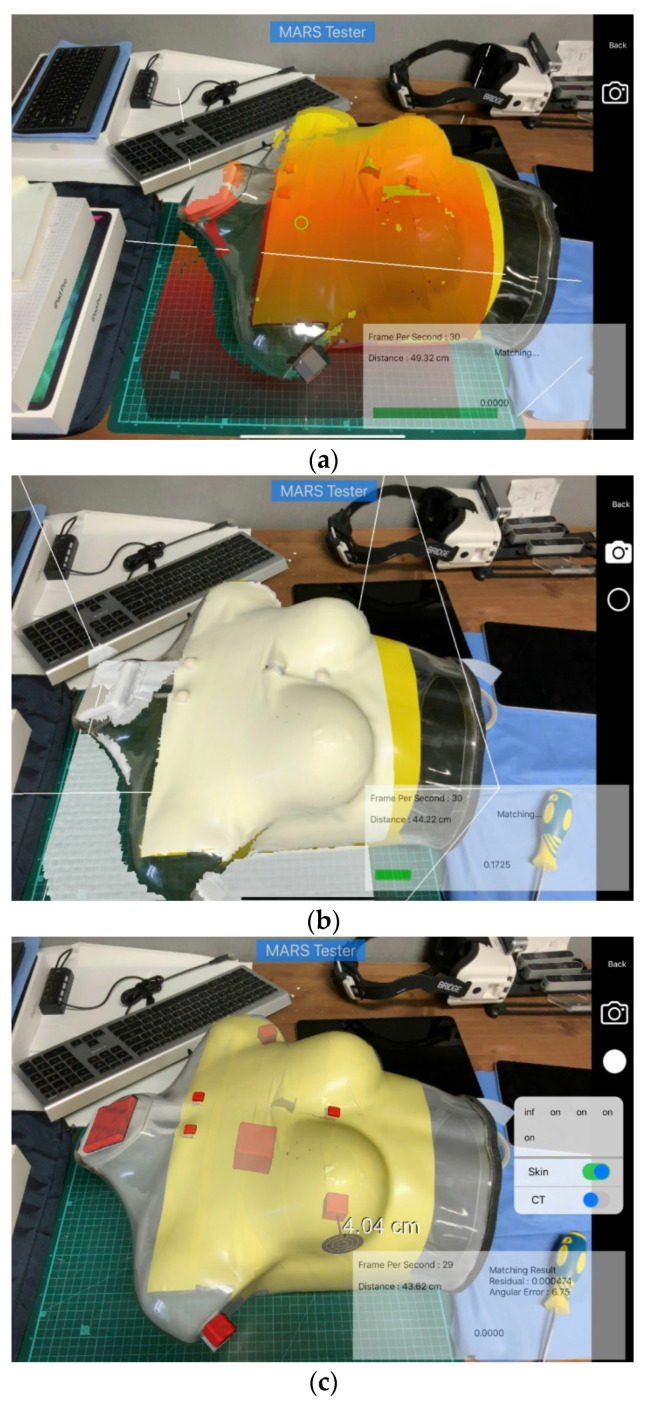
Phantom data registration and AR visualization. (**a**) 3D scan process. (**b**) Skin mesh AR visualization. (**c**) Cube AR visualization.

For the phantom data, a mannequin with a size similar to a human body was produced, and CT images were taken using the mannequin. Two cubes assumed to be lesions were placed inside the mannequin for qualitative evaluation of registration results, and five additional cubes were placed on the arm and neck. Experiments were performed using 20 scan data points taken from different angles and environments and the skin mesh generated through the proposed technique from the CT images taken. Figure 4 shows the results of registration and AR visualization using phantom data. Figure 4a shows the sight of taking the 3D scan, and Figure 4b,c are the results of AR visualization for the skin mesh and each cube after registration. It can be seen that the mannequin and AR show good agreement with high accuracy. The registration accuracy of the phantom data is presented in (Table 2), and the average registration accuracy is 3.44 ± 1.37 mm.

**Figure 5:**
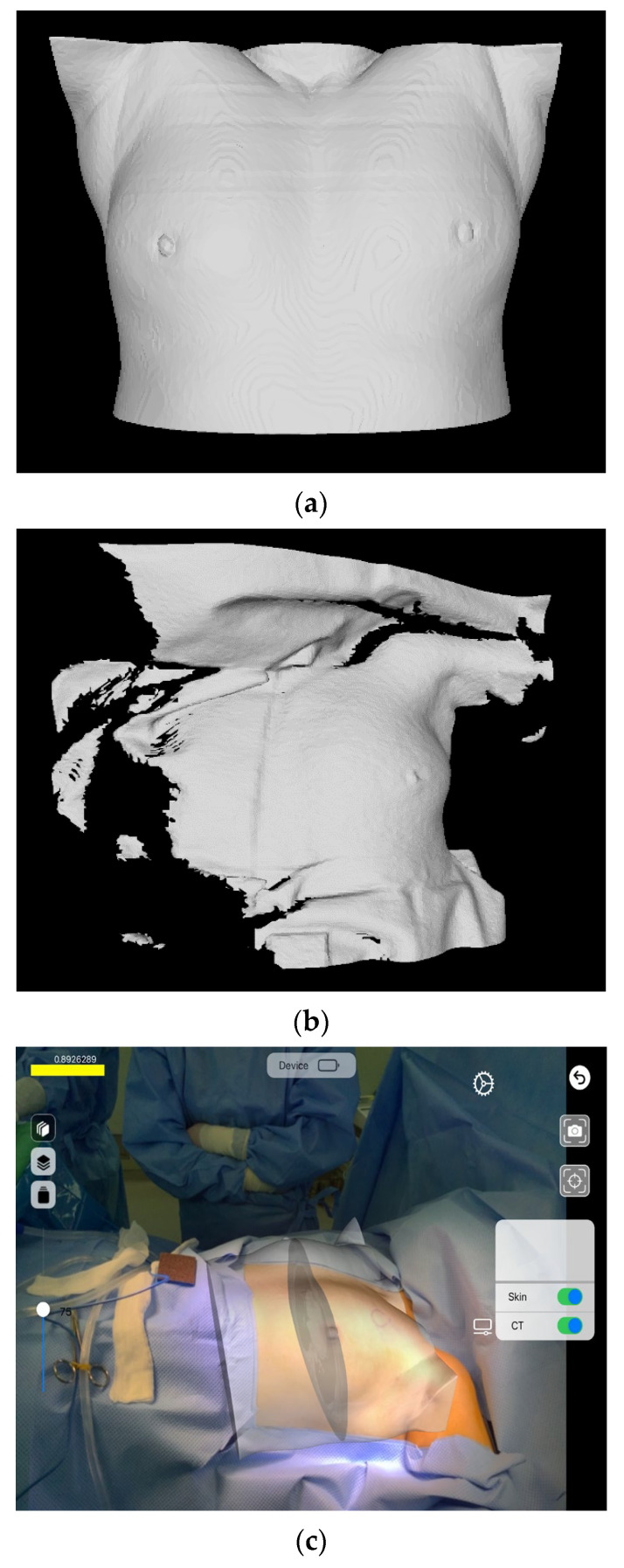
Results of patients’ data registration and AR visualization. (**a**) Skin mesh. (**b**) 3D scan result. (**c**) AR visualization of registration result.

As for patient data, CT images of 20 patients and their 3D scanning results were obtained before they underwent a surgical operation. $M_{c}$ was derived from each CT image, and $M_{p}$ was derived from each 3D scanning result. Subsequently, registration was conducted based on the derived results. Figure 5 shows the registration results. Figure 5a,b show $M_{c}$ and $M_{p}$, respectively, and Figure 5c shows the result of visualizing the registration results based on AR. Table 2 shows the accuracy of the registration of patient data. The mean registration error was calculated as 4.69 ± 1.95 mm.

## 4. Conclusions

In this study, we proposed a markerless AR surgical framework that provides 3D integrated visualization of the patient’s body by utilizing the 3D breast CT taken before surgery and the mesh obtained through a 3D scan of the patient. To perform registration of the 3D breast CT data with the mesh from the patient’s scan, contrast-based skin region segmentation was performed and a 3D skin mesh was generated. The skin mesh was smoothed by applying the Gaussian skin filter [20], and decimation was performed considering the mesh quality and computational load in the registration process. In addition, in order to consider the environment of the operating room, the vertices to be used for registration are selected by setting the ROI based on the nipple area obtained by gradient-based search. For registration, the first alignment was made by matching the centers of the two meshes, and more precise registration was performed using the ICP technique [12]. As a result of the experiment, the skin segmentation accuracy was 98.35 ± 0.71% in terms of DSC value, and the nipple detection error was 2.79 ± 1.54 mm. To evaluate registration accuracy, phantom data and patients’ data were used, and the errors were 3.44 ± 1.37 mm and 4.69 ± 1.95 mm, respectively.

The conventional AR surgical system involves attaching markers for high accuracy and using additional cameras for marker detection, which is not practical in clinical settings. Park et al. [4] applied AR in spine surgery for the spine with small deformation and showed an error of about 2.22 mm. However, this method requires 3–6 optical markers for registration and 2–3 infrared cameras for marker detection. Adagolodjo et al. [5] proposed an AR system for open liver surgery and showed an error of about 2.69 mm. However, after opening the abdominal cavity for registration, markers have to be attached to the liver, and the fewer the number of markers used, the lower the registration accuracy. This method also requires four infrared cameras to detect the markers.

The proposed method in this study showed that 3D AR visualization of medical data on the patient’s body is possible by using a single depth sensor without having to use markers. In addition, the proposed method allows the seamless provision of additional information by reflecting the registration factor onto the segmented lesion area. This indicates that the proposed method allows practical application in real-world clinical practice compared to the conventional marker-based methods.

As a limitation of this study, it has a limitation in that it does not consider changes in a patient’s body caused by surgical resection that occurs during surgery. Hence, further research will be conducted to obtain additional clinical data, analyze dynamic physical deformation simulation, and ultimately reflect the deformation that occurs during a surgical operation.

## Figures and Tables

**Table 1:** Accuracy of skin segmentation and nipple detection.

Case	Skin Segmentation Accuracy Measured by DSC (%)	Nipple Detection Accuracy (mm)
1	98.37	2.00
2	98.58	1.90
3	99.56	3.46
4	97.61	5.72
5	97.87	1.80
6	99.28	1.26
7	96.76	3.63
8	97.72	2.54
9	98.12	2.12
10	99.51	5.43
11	98.73	1.60
12	97.96	0.12
13	99.24	1.23
14	98.60	3.24
15	98.56	5.45
16	97.42	2.47
17	98.41	1.43
18	98.17	4.39
19	98.10	3.34
20	98.51	2.72

**Table 2:** Registration error with phantom and actual patient data.

Case	Phantom Data (mm)	Actual Patient Data (mm)
1	3.41	2.56
2	3.00	5.40
3	3.80	2.99
4	2.27	3.76
5	2.48	3.11
6	2.71	2.03
7	3.02	6.29
8	6.34	5.85
9	4.29	3.98
10	2.91	7.13
11	5.69	5.51
12	3.33	2.74
13	1.72	6.72
14	2.10	6.62
15	5.05	5.25
16	1.59	4.22
17	2.27	7.88
18	5.70	2.29
19	4.08	2.03
20	3.15	7.40

## Data Availability

Not applicable.
